# Citizen Contribution for Searching for Alternative Antimicrobial Activity Substances in Soil

**DOI:** 10.3390/antibiotics12010057

**Published:** 2022-12-29

**Authors:** Rosa Fernández-Fernández, Beatriz Robredo, Enrique Navajas, Carmen Torres

**Affiliations:** 1 Area of Biochemistry and Molecular Biology, Faculty of Science and Technology, University of La Rioja, 26006 Logroño, Spain; 2 Area of Didactic of Experimental Sciences, Faculty of Science and Technology, University of La Rioja, 26006 Logroño, Spain

**Keywords:** antimicrobial-producing bacteria, soil, antimicrobial resistance, MicroMundo

## Abstract

Antimicrobial resistance (AMR) is problematic worldwide, and due to the loss of efficiency of many antibiotics, the pressure to discover alternative antimicrobial molecules has increased. Soil harbors a great biodiversity and biomass of microorganisms, and many antibiotics are produced by soil microbiota. Therefore, soil is a promising reservoir to find new antimicrobial agents. In this respect, novel pedagogical strategies regarding the AMR global crisis have recently been developed in different countries worldwide. Highlighted is the service-learning project “MicroMundo” integrated in a global Citizen Science project called “Tiny Earth”. Hence, the present work aimed at determining the antimicrobial activity of soil bacteria, the biodiversity of the selected isolates as putative antimicrobial producers, and their antibiotic resistance profile. Moreover, through the MicroMundo project, we tried to illustrate the relevant link between science and education and the benefits of implementing service-learning methodologies to raise awareness of the AMR problem and to contribute to the search for new alternatives. A total of 16 teachers, 25 university students and 300 secondary school students participated in the search for antimicrobial activity on a collection of 2600 isolates obtained from a total of 130 soil samples analysed. In total, 132 isolates (5% of total tested) were selected as potential antimicrobial producers when two indicator bacteria were used (Escherichia coli and Staphylococcus epidermidis); the most frequent genus among these isolates was Bacillus, followed by Pseudomonas, Paenibacillus and Serratia. The antimicrobial activity (AA) of the 132 potential antimicrobial producers was studied in a second step against 15 indicator bacteria (of six genera and thirteen species, including relevant pathogens). Of the 132 potentially producing bacteria, 32 were selected for further characterization. In this respect, 18 isolates showed low AA, 12 isolates were considered as medium producers, and 2 highly antimicrobial-producing isolates were found (Brevibacillus laterosporus X7262 and Staphylococcus hominis X7276) showing AA against 80% of the 15 indicators tested. Moreover, 48% of the antimicrobial-producing bacteria were susceptible to all antibiotics tested. Due to citizen science, antimicrobial-producing bacteria of great interest have been isolated, managing to raise awareness about the problem of AMR.

## 1. Introduction

Antimicrobial resistance (AMR) has risen an awareness alarm, being one of the most urgent challenges for current medicine and society due to the emergence of multi-drug resistant (MDR) pathogens [1,2]. Concretely, the science community is especially concerned about the antimicrobial resistance associated with the ESKAPE pathogens (*Enterococcus faecium*, *Staphylococcus aureus*, *Klebsiella pneumoniae*, *Acinetobacter baumannii*, *Pseudomonas aeruginosa*, and *Enterobacter* species) [3].

The loss of efficacy of many antibiotics increases the pressure to identify new effective approaches [4]. In the last decade, intensive studies have looked at the potential of natural antibacterial molecules as next-generation therapeutics against pathogens [4,5]. Concretely, the ribosomally synthesized peptides of bacterial origin, also named as bacteriocins, are one of the most promising bioactive compounds with antimicrobial properties against other bacteria [5]. The ability to synthetize bioactive peptides is one of the oldest defensive mechanisms of microorganisms, and many microorganisms produce at least one bacteriocin [6]. These not essential secondary metabolites increase the bacterial chances of adaptation in a hostile environment, and they have been proposed as a good alternative to combat pathogens and MDR bacteria [5,7].

Soil harbors a great biodiversity and biomass of microorganisms [8,9]. Soil bacteria live in a crowded and highly competitive environment with limited resources and constantly changing conditions. There are many antibiotics produced by microorganisms isolated from soil, from penicillin, the first reported, to some new ones such as malacidins and teixobactin [10]. Actinomycetes, which are the most common bacteria in soil, produce 60% of antibiotics in clinical use [11]. Thus, the potential to find new antimicrobial compounds in this immense reservoir of microorganisms is enormous, and scientists are beginning to realize how little is known regarding soil microorganisms.

To address these problems, novel pedagogical strategies on the AMR global crisis have recently been developed in different countries worldwide. Among such strategies is “MicroMundo” [12], integrated in a global Citizen Science project on AMR called “Tiny Earth” (TE; https://tinyearth.wisc.edu/, accessed on 1 December 2022) originally implemented in 2012 in the United States, with “Small World Initiative” designation (SWI; http://www.smallworldinitiative.org/, accessed on 1 December 2022).

MicroMundo was developed in a service-learning environment, in which different educational levels are integrated [12]. The program seeks to raise awareness of the problem of AMR among students by participating in a creative research project that combines soil sample collection and laboratory work to discover new antimicrobial agents [13].

Hence, the present work aimed at determining the antimicrobial activity of bacteria of the soil, the biodiversity of the selected isolates as putative producers, and their antimicrobial resistance profile. Moreover, we tried to illustrate the relevant link between science and education and the benefits of implementing service-learning methodologies to raise awareness of AMR and to contribute to the search for new alternatives.

## 2. Material and Methods

### 2.1. MicroMundo: Service-Learning Methodology

During the 2020–2022 school years, the service-learning project called MicroMundo was developed in La Rioja region (Spain) through two scales of practical training, involving university and secondary education.

The initial step of the MicroMundo project was carried out at the university (University of La Rioja), taught by a qualified professor (SWIPI: Partner Instructor) to master students (n = 25) or biology secondary school teachers (n = 12) (SWITAs: technical assistants). The SWIPI trained the teaching abilities of SWITAs through periodic sessions, and at the end, they discussed the logistics of SWI actions to analyze the experimental results and to design data-recording sheets.

The second step was performed in secondary schools (La Rioja region) under the general supervision of SWIPI, using expendable material and equipment from the university. In this process, the 37 SWITAs (master students and secondary school teachers) and 299 students (SWISs) of 12 secondary schools were involved. The SWITAs trained the program to the SWISs through periodic sessions. They made up a total of 130 groups, each of whom analyzed a soil sample.

Practical work was divided into five 2 h sessions following the methodology previously explained [14]. Firstly, soil samples were diluted and grown on Tryptic Soy Agar (TSA) (Condalab, Madrid, Spain) plates for colony selection (20 isolates per sample). An antimicrobial activity test was performed with all 20 isolates obtained on each soil sample, using *Staphylococcus epidermidis* C2663 (Gram-positive bacteria) and *Escherichia coli* C408 (Gram-negative bacteria) as indicator microorganisms (Table S1). Indicator bacteria were inoculated in saline solution and spread as a lawn onto TSA plates. Then, bacteria to be tested for antimicrobial activity production were transferred with a sterile toothpick. Plates were evaluated by students after 24 h, and isolates with putative inhibition halloes were selected in this initial screening and transferred to the university for verification and further characterization (second screening).

### 2.2. Second Screening of Antimicrobial Activity by the Spot-on-Lawn Method

The selected strains with potential antimicrobial activity obtained in the first screening at school level were further analyzed and characterized in a second screening process at the university. For that purpose, these strains were tested by the *spot-on-lawn* method against 15 indicator strains of 6 different genera and 13 species, including relevant pathogens (Supplementary Table S1). Bacteria were grown in brain heart infusion (BHI) agar (Condalab, Madrid, Spain) for 24 h at 37 °C. To prepare test plates, a suspension of the indicator strain was prepared in a tube of 3 mL of sterile saline solution to obtain a turbidity of 0.5 MacFarland, and it was spread with a sterile swab in 0.3% yeast extract-supplemented solid Tryptic Soy Agar (TSA) plate (Condalab, Madrid, Spain). Then, each fresh solid culture of bacterium to be tested for antimicrobial activity was transferred with a sterile toothpick to the agar plates seeded with each of the 15 indicators tested. Plates were incubated at 37 °C for 24 h to evaluate the halos of inhibition. Isolates were considered antimicrobial producers (AP) when they showed a clear and sharp inhibition zone against at least one of the 15 indicator isolates. Depending on the antimicrobial activity (hallo of inhibition), it was expressed as + (<3 mm), ++ (3 < x < 10 mm), or +++ (>10 mm).

### 2.3. Bacterial Identification

Isolates with potential antimicrobial capacity obtained in the first screening (*n* = 132) were identified by matrix-assisted laser desorption/ionization time of flight (MALDI-TOF) mass spectrometry, using the standard protein extraction protocol recommended by the commercial device of Bruker Daltonics, Bremen, Germany. The antimicrobial-producing isolates of interest that could not be identified by MALDI-TOF were identified by amplification and sequencing of the 16S rDNA gene, using the following primer sequences (F-GTGCCAGCAGCCGCGGTAA, R-AGACCCGGGAACGTATTCAC) and PCR conditions: 94 °C, 2 min; 28 cycles (94 °C, 30 s; 45 °C, 1 min; 72 °C, 1 min) and final 72 °C, 7 min [15].

### 2.4. Antibiotic Susceptibility Testing of Antimicrobial-Producing Strains

The antibiotic susceptibility profile was determined in the antimicrobial-producing isolates verified in the second screening, by the disk diffusion test in Mueller Hinton (MH) agar (Condalab, Madrid, Spain). The antibiotics used for that purpose differed depending on the bacterial genera and are indicated in Supplementary Table S2. The EUCAST guidelines [16] were used for the antibiotic susceptibility testing, and *Staphylococcus* spp. breakpoints were used for Gram-positive bacteria, except for *Bacillus* spp., for which EUCAST indicates a specific breakpoint for some antimicrobial agents [16]. In the case of Gram-negative bacteria, the breakpoints of enterobacterales and *Pseudomonas* were used, depending on the genera.

### 2.5. Diversity of Antimicrobial-Producing Bacteria and Statistics

The 130 soil samples were grouped based on the geographic coordinates of the collection site. In this way, 5 geographical zones were distinguished: La Rioja East, La Rioja Central, La Rioja West, Logroño and Outside (Supplementary Figure S1). Statistics comparison between the antimicrobial-producing bacteria detected by the first screening and the non-antimicrobial-producing isolates included in the 5 established clusters was performed using the Fisher test, and significant differences were considered for *p* < 0.05. Moreover, diversity and statistical analyses were carried out considering each antimicrobial-producing isolate as an independent item. Isolates that could not be identified were excluded for diversity analysis; thus, a collection of 104 isolates was included. Renyi profile was performed to compare alpha diversity between the 5 clusters. The Renyi profile was used to compare the species diversity among zones with vegan 2.6–2 package from R (4.2.1). The averages of the Renyi profile values were calculated using rarefied samples. Significant differences in these values were assessed with an ANOVA test. Tukey’s post hoc test was used to identify differences between zones. All analyses were carried out with R, version 4.1.2.

## 3. Results

A total of 130 soil samples were analyzed at school level, and 2600 isolates were obtained (20 isolates/sample) and tested in a first screening for antimicrobial activity; 132 isolates showed potential inhibitory capacity in the first screening test performed in the school and using only two indicator bacteria (*S. epidermidis* and *E. coli*). Statistical differences were not observed between antimicrobial-producing and non-antimicrobial-producing isolates based on their geographical location.

**Figure 1:**
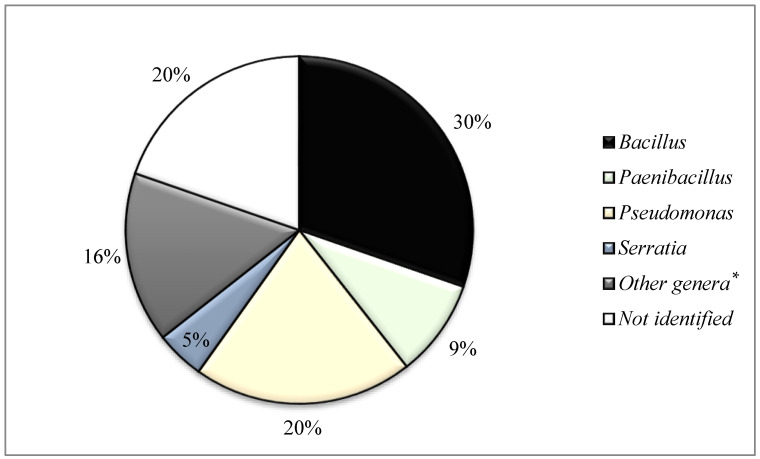
Diversity at genus level of the antimicrobial-producing bacteria isolated from soil samples in the first initial screening. * Other genera: include isolates of genera with less than six representants; see Table 1.

Identification by MALDI-TOF at genus level of these 132 putative antimicrobial-producing isolates detected in soil samples revealed 19 genera and 48 species with the following microbial diversity (number of isolates): *Acinetobacter* (1), *Arthrobacter* (2), *Bacillus* (40), *Bradybacterium* (1), *Brevibacillus* (1), *Enterobacter* (3), *Escherichia* (2), *Klebsiella* (1), *Microbacterium* (2), *Micrococcus* (1), *Paenibacillus* (12), *Pseudomonas* (27), *Staphylococcus* (2), *Serratia* (6), *Stenotrophomonas* (1), *Streptomyces* (1), *Olivibacter* (1), *Variovarax* (1) and *Viridibacillus* (1). Moreover, 26 out of the 132 isolates (19.7%) could not be identified by MALDI-TOF mass spectrometry (Table 1). Diversity at genera level is represented in Figure 1, where the number of isolates of *Bacillus*, *Paenibacillus*, *Pseudomonas*, *Serratia* as well as those unidentified are shown. Genera with low numbers of representants were considered together in a group called “Others” (n = 19 isolates). A high prevalence of the genus *Bacillus* followed by *Pseudomonas*, *Paenibacillus* and *Serratia* (all isolates belong to the species *S. plymuthica*) is of note.

Renyi profiles allowed us to differentiate clusters according to the diversity of potential producers (Figure S1). The community with an overlapping diversity profile was considered the most diverse. Thus, the decreasing ranking of clusters was as follows: La Rioja Central, La Rioja West and Logroño (same alpha values), La Rioja East and Outside (Figure S2). This revealed that antimicrobial-producing isolates of La Rioja Central had the best distribution of richness (number of different genera/species) and abundance (number of isolates of each genus/species detected) (Table S3 and Figure S2).

### 3.1. Verification of Antimicrobial Activity of Antimicrobial-Producing Bacteria in a Second Screening Process against 15 Indicator Bacteria

The 132 isolates obtained in the first screening with potential antimicrobial activity production were tested at the university in a second screening by the *spot-on-lawn* method against 15 indicator bacteria. Then, 32 out of the 132 tested isolates were finally selected for presenting clear antimicrobial activity after several repetitions against at least one indicator bacteria (Table 2). Identification of 29 out of 32 isolates was obtained by MALDITOF, and the remaining 3 (X7264, X7265 and X7266) were identified by amplification and sequencing of the 16S rDNA gene. Twenty-three of the 32 antimicrobial-producing isolates were Gram-positive (15 species and 6 genera), and nine were Gram-negative (6 species and 3 genera), and they were further characterized. Most of antimicrobial antimicrobial-producing bacteria were of the genera *Bacillus* (43.8%) and *Pseudomonas* (21.9%) (Table 2).

The most susceptible indicators detected in this study with antimicrobial-producing isolates were the following: *S. epidermidis*, *M. luteus*, and methicillin-resistant and -susceptible *S. aureus* (MRSA and MSSA, respectively) (Table 2 and Table 3). Seven Gram-positive producing isolates showed antimicrobial activity against the Gram-negative indicators used (*E. coli* and *P. aeruginosa*). It is to note the high inhibition produced by both Gram-positive and Gram-negative antimicrobial-producing bacteria against the indicators of the genus *Staphylococcus*, including methicillin-resistant staphylococci. In addition, three Gram-positive antimicrobial-producing isolates (*B. mycoides* X7258; *B. laterosporus* X7262 and *S. hominis* X7276) inhibited the indicator *L. monocytogenes*, a relevant pathogen in the food industry. Finally, excluding the species *E. cecorum* (with 48% of inhibition), *Enterococcus* was the most resistant indicator genus, only inhibited by Gram-positive antimicrobial-producing isolates, as expected (Table 2 and Table 3).

**Figure 2:**
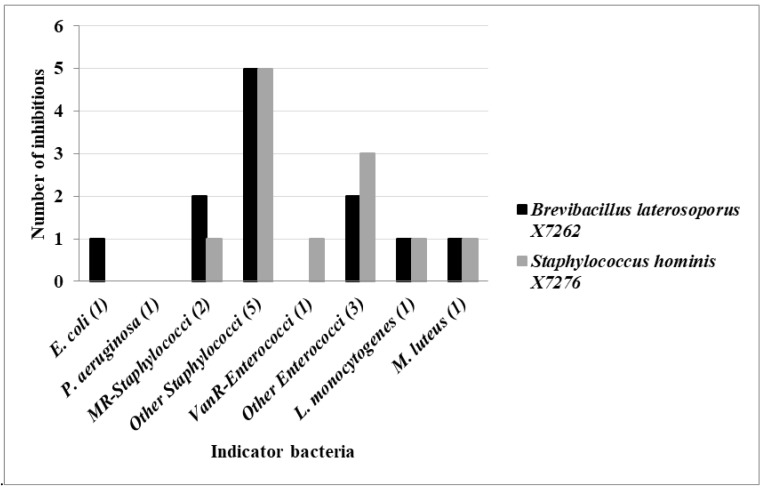
Number of indicator bacteria inhibited by the two highly producing isolates (*Brevibacillus laterosporus* X7262 and *Staphylococcus hominis* X7276).

Three levels were differentiated regarding production based on the percentages of indicator bacteria inhibited by the antimicrobial-producing isolates: low (<35%), medium (from 35 to 70%) and high (>70%). In this respect, 18 isolates showed low antimicrobial activity, 12 isolates were considered as medium producers, and 2 isolates were found to be high producers with antimicrobial activity against 80% of the indicators tested (*Brevibacillus laterosporus* X7262 and *Staphylococcus hominis* X7276) (Table 2). Neither of the two highly producing isolates were active against *P. aeruginosa,* but *B. laterosporus* X7262 inhibited *E. coli*, the other Gram-negative indicator strain. On the other hand, both highly producing strains showed antimicrobial activity against methicillin-resistant and -susceptible (MR and MS) staphylococci (from 90% to 100% of inhibition), *L. monocytogenes* and *M. luteus.* The *S. hominis* X7276 strain revealed antimicrobial activity against all *Enterococcus* isolates used as indicators (100%), and *B. laterosporus* X7262 inhibited 50% of them (Figure 2).

### 3.2. Antibiotic Resistance Phenotype of the Antimicrobial-Producing Isolates

In total, 48% of the 32 antimicrobial-producing isolates showed susceptibility to all the antibiotics tested. Considering Gram-positive isolates, resistance was mostly detected for cefoxitin (22%) and penicillin and tobramycin (17%). Meropenem/imipenem and ciprofloxacin resistance were also found among *Bacillus* isolates. Four Gram-positive producing isolates (17%) were multidrug resistant (MDR). With respect to Gram-negative, 33% of them were susceptible to all the antibiotics tested (all belonging to *Pseudomonas* genus). Four *Pseudomonas* spp. showed resistance to ticarcillin, and two of them were also resistant to aztreonam. Moreover, two Gram-negative isolates were resistant to ampicillin and cefoxitin, one of them being MDR (*Olivibacter* X7265) (Table 4).

## 4. Discussion

Soil contains a highly diverse collection of bacteria, making it a very attractive starting point for efforts to discover molecules with antimicrobial activity [17]. In this sense, the present work carried out a massive soil sampling due to the citizen collaboration of professors, teachers, university students and secondary education students, under the MicroMundo project.

Therefore, from a collection of 2600 bacteria, 132 putative antimicrobial producers were obtained in the first screening, which represent 5% of the total isolates recovered. When processing these samples in the laboratory during the second screening, 100 producers were lost, probably due to the stricter criteria of antibacterial effect verification at the university, considering only clear zones of inhibition as putative markers of bacteriocins. However, many other antimicrobial substances have been described apart from antimicrobial peptides with different phenotypes of inhibition halos not considered for this study. On the other hand, bacteriocins are known to be produced in response to signals received from a potential competitor, which then elicits an antagonistic response [18]. Therefore, in this study, the 32 isolates, which showed constant antimicrobial activity throughout the second screenings carried out, were selected for their subsequent characterization.

This work provides information on the soil biodiversity of bacteria with potential inhibitory capacity. Renyi profiles of La Rioja zones reveal a higher diversity in La Rioja Central, although a higher number of antimicrobial-producing isolates among the 132 firstly identified were detected among Logroño samples. In this regard, *Bacillus* (30%) and *Pseudomonas* (20%) were the most predominant genera, in accordance with what was observed by Huang et al., 2021 [19]. However, other genera were found in this study, such as *Paenibacillus* or *Serratia*. These results highlight the potential of soil as a reservoir of bacteria that produce antimicrobial agents; thus, further characterization of isolates could be of interest.

In recent years, bacteria such as *Pseudomonas* spp. and *Bacillus* spp. have been studied and used as biological control agents for plant diseases [20,21], including the antibiosis mechanism for competition for nutrients and niches [22]. *Bacillus* is a genus well known as a producer of antibacterial substances such as lipopeptides, phenols, proteases, and bacteriocins [23]. Species of the genus *Pseudomonas* produce several secondary metabolites that affect other bacteria, fungi, or predators of nematodes and protozoa, such as bacteriocins, ranging from small microcin to large tailocin [24].

Thus, as expected, 14 *Bacillus* spp. and 7 *Pseudomonas* spp. out of the 32 bacteria finally selected as clear producers of antimicrobial substances were identified in this work. According to the *spot-on-lawn* results, higher activity was found against Gram-positive indicator bacteria than against Gram-negative indicator bacteria, being the *Staphylococcus* genera, (including MR-*Staphylococci*), the most susceptible indicator bacteria. It is widely known that most microbial metabolites have specific antimicrobial potential, and they act at the target sites [2]. Seven Gram-positive isolates showed antimicrobial activity against the Gram-negative indicators used. In addition, *Brevibacillus laterosporus* X7262 and *Staphylococcus hominis* X7276 stand out as high producers, which show antimicrobial activity against MS-staphylococci, *L. monocytogenes* and *M. luteus*.

*Brevibacillus laterosporus* is an aerobic, spore-forming, entomopathogenic microorganism commonly isolated from soil. Some strains have potential activity as biological control agents [25]. In addition, several applications of this bacterium as a biological control agent have been described, highlighting the high toxicity against mosquito larvae among other insects and the activity that promotes growth and improves productivity in bee colonies [26,27,28].

As for *S. hominis*, it is a normal skin commensal coagulase negative staphylococci (CoNS) described as a bacteriocin producer such as hominicin [29] and nukacin KQU-131 [30], among others. Moreover, recent studies have detected bacteriocin-like-producing staphylococci of environmental origin, including *S. hominis* [31]. Due to their high tolerance to an acidic environment, the resistance to bile, and the capacity to adhering to an epithelial cell line, *S. hominis* has been proposed as a good candidate for probiotic treatments against *S. aureus* [32]. In this sense, Nakatsuji et al., 2017 [33], reported that human *Staphylococcus* commensal species produce antimicrobial peptides that protect us against pathogens that control skin microbiota imbalances, and they demonstrated that a personalized probiotic CoNS cream could alleviate the symptoms of skin dysbiosis such as atopic dermatitis.

In short, advanced and combinatorial therapies that include antibiotics or new molecules with antimicrobial activity could be used as an alternative solution to combat AMR from a biotechnological and biomedical perspective and to solve problems in the agriculture and food industries, among others [34]. The one-health perspective makes clear the need for an ecosystem union to achieve improved objectives in the problem of AMR. Citizens must be integrated into this system, knowing the problem of the urgent need to find antimicrobial molecules, becoming aware of it, and contributing to research through this type of citizen science and service-learning initiatives such as MicroMundo.

## Figures and Tables

**Table 1:** Genus and species identification of the 132 potential antimicrobial-producing isolates obtained in the first screening and those 32 isolates verified in the second screening.

Genus	Species	Number of Isolates First Screening	Number of Isolates Second Screening
*Acinetobacter*	*Acinetobacter radioresistens*	1	
*Arthrobacter*		2	
	*Arthrobacter citreus*	1	1
	*Arthrobacter ilicis*	1	
*Bacillus*		40	
	*Bacillus marisflavi*	1	
	*Bacillus atrophaeus*	2	2
	*Bacillus cereus*	6	1
	*Bacillus cibi*	1	
	*Bacillus megaterium*	4	1
	*Bacillus mycoides*	4	1
	*Bacillus pumilus*	7	5
	*Bacillus safensis*	2	2
	*Bacillus simplex*	3	
	*Bacillus thuringiensis*	2	
	*Bacillus weihenstefanensis*	3	
	*Bacillus* spp.	5	2
*Bradybacterium*	*Bradybacterium* spp.	1	1
*Brevibacillus*	*Brevibacillus laterosoporus*	1	1
*Enterobacter*	*Enterobacter cloacae*	3	
*Escherichia*	*Escherichia coli*	2	
*Klebsiella*	*Klebsiella aerogenes*	1	1
*Microbacterium*	*Microbacterium arborescensens*	2	1
*Micrococcus*	*Micrococcus luteus*	1	
*Paenibacillus*		12	
	*Paenibacillus amylolyticus*	6	
	*Paenibacillus apiarus*	2	2
	*Paenibacillus gluconolyticus*	1	
	*Paenibacillus lautus*	1	
	*Paenibacillus polymyxa*	1	1
	*Paenibacillus xylanilyticus*	1	
*Pseudomonas*		27	
	*Pseudomonas brasicacearum*	2	
	*Pseudomonas brenneri*	1	
	*Pseudomonas caricapapayae*	1	1
	*Pseudomonas chlororaphis*	3	1
	*Pseudomonas kilonensis*	5	3
	*Pseudomonas koreensis*	2	
	*Pseudomonas mandelii*	1	
	*Pseudomonas mosselii*	1	
	*Pseudomonas putida*	1	
	*Pseudomonas savastanoi*	1	
	*Pseudomonas thivervalensis*	2	
	*Pseudomonas umsongensis*	1	
	*Pseudomonas* spp.	6	2
*Staphylococcus*	*Staphylococcus hominis*	2	1
*Serratia*	*Serratia plymuthica*	6	
*Stenotrophomonas*	*Stenotrophomonas rhizophila*	1	
*Streptomyces*	*Streptomyces avidinii*	1	1
*Olivibacter*	*Olivibacter soli*	1	1
*Variovorax*	*Variovorax paradoxus*	1	
*Viridibacillus*	*Viridibacillus arenosi*	1	
Not identified		26	
Total		132	32

**Table 2:** Activity profile of the 32 antimicrobial-producing isolates against the 15 indicator bacteria tested.

	Antimicrobial Activity ^a^ on the Indicator Bacteria ^b^															
Producing Isolate	*E. coli*	*P. aeruginosa*	MRSA	MSSA	MRSP	MSSP	*S. delphini*	*S. sciuri*	*S. epidermidis*	*E. faecalis*	*E. faecium* ^c^	*E. cecorum*	*E. gallinarum*	*L. monocytogenes*	*M. luteus*	No (%)
*A. citreus* X7246				+					+						+	3 (20)
*B. cereus* X7247			+			+			+			+			+	5 (33)
*B. safensis* X7248			+			+			+			+			+	5 (33)
*B. safensis* X7249	+		+			+			++			+			+	6 (40)
*B. pumilus* X7250	+		+	+		+	+		+			+			+	8 (53)
*B. atrophaeus* X7251			+			+			+			+			+	5 (33)
*B. atrophaeus* X7252	+		+	+		+			+			+			+	7 (47)
*Bacillus* spp. X7253				+					+						+	3 (20)
*Bacillus* spp. X7256.			+	+		+	+	+	+			+			+	8 (53)
*B. pumilus* X7254			+			+	+		+			+			+	6 (40)
*B. megaterium* X7255				+					+						+	3 (20)
*B. pumilus* X7257		+							+						+	3 (20)
*B. mycoides* X7258			+	+		+	+	+	+					+	+	8 (53)
*B. pumilus* X7259			+	+	+	+	+		+			+			+	8 (53)
*B. pumilus* X7260			+	+	+	+	+		+						+	7 (47)
*Bradybacterium* spp. X7261				+					+							2 (13)
*B. laterosoporus* X7262	+		+	+	+	+	+	+	+	+		+		+	+	12 (80)
*M. arborescensens* X7263			+	+	+	+	+		+						+	7 (47)
*P. apiarus* X7264	+		+				+		+						+	5 (33)
*P. apiarus* X7267			+						+						+	3 (20)
*P. polymyxa* X7268	+		+	+		+	+		+			+			+	8 (53)
*S. hominis* X7276			+	+		+	+	+	+	+	+	+	+	+	+	12 (80)
*S. avidinii* X7277			+	+					+						+	4 (27)
Number of inhibitions (%)	6 (26)	1 (4)	18 (78)	15 (65)	4 (17)	15 (65)	11 (48)	4 (17)	23 (100)	2 (9)	1 (4)	12 (52)	1 (4)	3 (13)	22 (92)	
*O. soli* X7265				+					+						+	3 (20)
*K. aerogenes* X7266	+			+		+										3 (20)
*P. kilonensis* X7269			+	+	+	+	+	+	+++						+	8 (53)
*P. kilonensis* X7270			+				+		+++						+	4 (27)
*P. kilonensis* X7271			+	+					+						+	4 (27)
*Pseudomonas* spp. X7272			+	+					+							3 (20)
*Pseudomonas* spp. X7273			+	+					+							3 (20)
*P. chlororaphis* X7274	+	+	+	+	+		+		+						+	8 (53)
*P. caricapapayae* X7275	+	+		+					+						+	5 (33)
Number of inhibitions (%)	3 (33)	2 (22)	6 (67)	8 (89)	2 (22)	2 (22)	3 (33)	1 (11)	8 (89)	0 (0)	0 (0)	0 (0)	0 (0)	0 (0)	6 (67)	

^a^ Depending on the antimicrobial activity, it was expressed as + (<3 mm), ++ (3 < x < 10 mm), and +++ (>10 mm). Only positive results are indicated. ^b^ Abbreviations: MRSA, methicillin-resistant *S. aureus*; MSSA, methicillin-susceptible *S. aureus*; MRSP; methicillin-resistant *S. pseudintermedius*; MSSP, methicillin-susceptible *S. pseudintermedius*. ^c^ Vancomycin resistant strain.

**Table 3:** Antimicrobial activity profile of the 32 antimicrobial-producing isolates obtained in the second screening against 15 indicator bacteria (number of isolates).

	Number of Positive Results of the Antimicrobial-Producing Isolates against the Following Indicator Bacteria:						
Producing Isolates	*E. coli* (1)	*P. aeruginosa* (1)	MR-*Staphylococcus* ^a^ (2)	MS-*Staphylococcus* ^a^ (5)	*Enterococcus* (4)	*L. monocytogenes* (1)	*M. luteus* (1)
*A. citreus* (1)				2			1
*Bacillus* spp. (14)	3	1	13	41	9	1	14
*Bradybacterium* spp. (1)				2			
*B. laterosoporus* (1)	1		2	5	2	1	1
*M. arborescensens* (1)			2	4			1
*Paenibacillus* spp. (3)	2		3	7	1		3
*S. hominis* (1)			1	5	4	1	1
*S. avidinii* (1)			1	2			1
*O. soli* (1)				2			1
*K. aerogenes* (1)	1			2			
*Pseudomonas* spp. (7)	2	2	8	18			5

^a^ Abbreviations: MR, methicillin resistant; MS, methicillin susceptible.

**Table 4:** Antimicrobial resistance phenotype of the selected 32 antimicrobial-producing isolates obtained in the second screening.

Type of Bacteria	Number of Isolates	Genus	Species ^a^	Antimicrobial Resistance Phenotype ^b^
Gram-positive	1	*Arthrobacter*	*A. citreus*	Susceptible
	7	*Bacillus* spp.	*B. pumilus*^2^, *B. safensis*, *B. megaterium*, *B. mycoides*, *Bacillus* spp. ^2^	PEN ^3^-FOX ^4^-MER ^3^-IMI ^2^-S ^2^-TOB ^3^-CLI -GEN-SXT-CIP ^3^
	7	*Bacillus* spp.	*B. pumilus*^3^, *B. cereus*, *B. artrophaeus*^2^, *B. safensis*	Susceptible ^7^
	1	*Bradybacterium*	*Bradybacterium* spp.	Susceptible
	1	*Brevibacillus*	*B. laterosporus*	Susceptible
	1	*Microbacterium*	*M. arborescensis*	Susceptible
	2	*Paenibacillus*	*P. apiarus* ^2^	PEN-FOX-TOB
	1	*Paenibacillus*	*P. polymyxa*	Susceptible
	1	*Staphylococcus*	*S. hominis*	Susceptible
	1	*Streptomyces*	*S. avidinii*	Susceptible
Gram-negative	1	*Klebsiella*	*K. aerogenes*	AMP-AMC-FOX
	1	*Olivibacter*	*O. soli*	AMP-FOX-CTX-CAZ-C-TOB
	4	*Pseudomonas*	*P. chlororaphis*, *P. caricapapayae*, *P. kilonensis*^2^	TIC ^4^-ATM ^2^
	3	*Pseudomonas*	*Pseudomonas* spp. ^2^, *P. kilonensis*	Susceptible

^a^ Superscript 2, 3 indicates the number of isolates of each species. ^b^ Superscript 2, 3, 4, 7 indicates the number of isolates with a specific characteristic, when more than one.

## Supplementary Materials

The following supporting information can be downloaded at: https://www.mdpi.com/article/10.3390/antibiotics12010057/s1, Figure S1: Coordinates of all soil samples analyzed in this study. Each cluster is differentiated by colors; Figure S2: Renyi diversity profile of the antimicrobial-producing microbial community included in each of the following zones: La Rioja Central, La Rioja East, La Rioja West, Logroño and Outside; Table S1: Characteristics of the 15 indicator bacteria used in this study for the screening of antimicrobial activity production; Table S2: Antibiotics used for disk-diffusion test in Gram-positive and -negative antimicrobial-producer isolates (EUCAST); Table S3: Number of isolates included in each of the established regions based on their sampling location.
