# Supplementary material for: Citizen Contribution for Searching for Alternative Antimicrobial Activity Substances in Soil
Source: Antibiotics (Basel). 2022 Dec 29;12(1):57. doi: 10.3390/antibiotics12010057 (PMC9854653; doi:10.3390/antibiotics12010057)
Supplement: Supplementary file 1 [file antibiotics-12-00057-s001.zip › antibiotics-2104603-supplementary.pdf]

Supplemental material

Table S1. Characteristics of the 15 indicator bacteria used in this study for the screening of antimicrobial activity production.

Specie (no of strains)	Strain	Origin	Relevant Antimicrobial Resistance Phenotype/Genotype
***Staphylococcus aureus* (2)**	C1570<br>ATCC29213	Human	Methicillin/*mecA*
***Staphylococcus delphini* (1)**	C9459	Wild animal	
***Staphylococcus pseudintermedius* (2)**	C2381<br>C3468	Pet<br>Pet	Methicillin/*mecA*
***Staphylococcus epidermidis* (1)**	C2663	Human	Methicillin/*mecA* and Linezolid
***Staphylococcus sciuri* (1)**	C9780	Wild animal	
***Enterococcus faecalis* (1)**	ATCC29212		
***Enterococcus faecium* (1)**	C2321		Vancomycin/*vanA*
***Enterococcus gallinarum* (1)**	C2310		
***Enterococcus. cecorum* (1)**	X3809		
***Listeria monocytogenes* (1)**	CECT4032		
***Micrococcus luteus* (1)**	C157		
***Escherichia coli* (1)**	ATCC25922		
***Pseudomonas aeruginosa* (1)**	C3282		

Table S2. Antibiotics used for disk-diffusion test in Gram-positive and Gram-negative antimicrobial producer isolates (EUCAST).

Type of bacteria	Genera	Antibiotic	Abbreviation
Gram-positive	*Arthrobacter*	Penicillin	PEN
	Bacillus	Cefoxitin	FOX
	Bradybacterium	Imipenem	IMI
	Brevibacillus	Meropenem	MER
	Microbacterium	Vancomycin	VAN
	Paenibacillus	Erythromycin	ERY
	Staphylococcus	Clindamycin	CLI
	Streptomyces	Tetracycline	TET
		Gentamicin	GEN
		Tobramycin	TOB
		Streptomycin	S
		Trimethoprim-sulfamethoxazole	SXT
		Ciprofloxacin	CIP
		Chloramphenicol	C
		Linezolid	LZD
Gram-negative	*Klebsiella*	Ampicillin	AMP
	Olivibacter	Amoxicillin-clavulanic acid	AMC
		Cefoxitin	FOX
		Cefotaxime	CTX
		Ceftazidime	CAZ
		Imipenem	IMI
		Tetracycline	TET
		Ciprofloxacin	CIP
		Trimethopim-sulfamethoxazole	SXT
		Gentamicin	GEN
		Tobramycin	TOB
		Chloramphenicol	C
	Pseudomonas	Ticarcillin	TIC
		Levofloxacin	LEV
		Meropenem	MER
		Aztreonam	AZT
		Ceftazidime	CAZ
		Ciprofloxacin	CIP

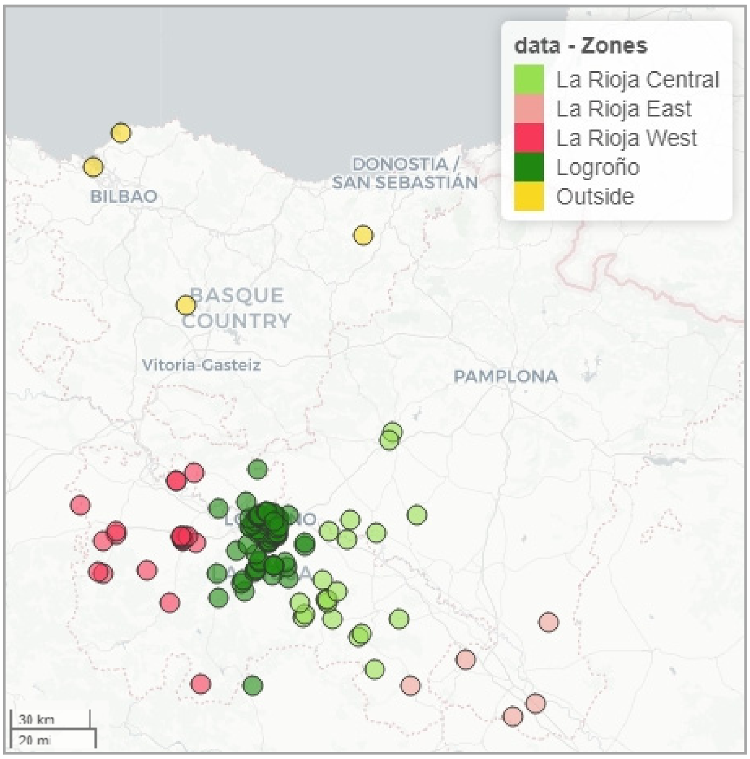

Figure S1. Coordinates of all soil samples analysed in this study. Each cluster is differentiated by colours.

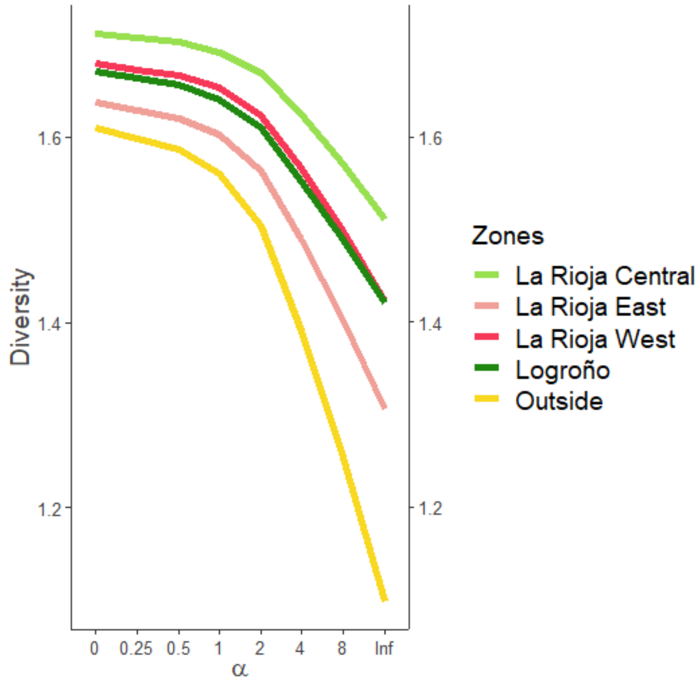

Figure S2. Renyi diversity profile of the antimicrobial-producing microbial community included in each of the following zones: La Rioja Central, La Rioja East, La Rioja West, Logroño and Outside.

Table S3. Number of isolates included in each of the established regions based on their sampling location.

	Total		Non-producing isolates		Producing isolates			
Cluster	Total isolates	No of samples	Non-producing isolates	No of samples	Producing isolates	No of samples	No of different Genera	No of different Species
La Rioja Central	400	20	378	12	22	8	7	12
La Rioja East	100	5	93	4	7	1	3	7
La Rioja West	420	21	400	12	20	9	4	11
Logroño	1580	79	1506	58	74	21	12	26
Outside	80	4	71	2	9	2	2	5
Total	2580	129	2448	88	132	41		
